# Transcriptome dataset of HEK293T cells depleted of one of the subunits of the DNA-PK complex: Ku70, Ku80 or DNA-PKcs

**DOI:** 10.1016/j.dib.2021.107596

**Published:** 2021-11-19

**Authors:** Andrey Anisenko, Olga Shadrina, Irina Garanina, Marina Gottikh

**Affiliations:** a Chemistry Department, Lomonosov Moscow State University, Moscow 199991, Russia; b Belozersky Institute of Physico-Chemical Biology, Lomonosov Moscow State University, Moscow 119992, Russia; c Department of Boiengineering and Bioinformatics, Lomonosov Moscow State University, Moscow 199991, Russia; d Federal Research and Clinical Center of Physical-Chemical Medicine of Federal Medical Biological Agency, Moscow 119435, Russia

**Keywords:** Ku heterodimer, DNA-dependent protein kinase, Ku70, Ku80, DNA-PKcs, NHEJ-related proteins

## Abstract

DNA-PK is a heterotrimeric complex that consists of Ku70 (XRCC6), Ku80 (XRCC5) and DNA-PKcs (PRKDC) subunits. The complex is a major player in the repair of DNA double strand break (DSB) via the non-homologous end joining (NHEJ) pathway. This process requires all DNA-PK subunits, since Ku70/Ku80 heterodimer firstly binds to DNA ends at DSB and then recruits DNA-PKcs. Recruitment of the DNA-PKcs subunit to DSB leads to phosphorylation events near DSB and recruitment of other NHEJ-related proteins that restore DNA integrity. However, today a lot of evidence demonstrates participation of the DNA-PK components in other cellular processes, e.g. telomere length maintenance, transcription, metabolism regulation, cytosolic DNA sensing, apoptosis, cellular movement and adhesion. It is important to note that not all the subunits of the DNA-PK complex are necessary for these processes, and the largest number of independent functions has been shown for the Ku70/Ku80 heterodimer and especially the Ku70 subunit. To better understand the role of each DNA-PK subunit in the cell life, we have analyzed transcriptome changes in HEK293T cells depleted of Ku70, Ku80 or DNA-PKcs using NGS-sequencing. Here, for the first time, we present the data obtained from the transcriptome analysis.

## Specifications Table

**Table:** 

Subject	Biological sciences
Specific subject area	Omics: Transcriptomics
Type of data	Tables, Figure
How data were acquired	Illumina HiSeq 1500
Data format	Raw sequences (FASTQ)
Parameters for data collection	RNA sequencing was performed for HEK293T cells or HEK293T with monoallelic knockout of Ku70, Ku80 and DNA-PKcs genes (Ku70+/-, Ku80+/-, DNA-PKcs+/-) treated with control siRNA or siKu70, siKu80 and siDNA-PKcs to additionally reduce the level of subsequent proteins.
Description of data collection	The samples collected for transcriptome analysis were immediately frozen in liquid nitrogen and stored at -70°C. For each sample, the experiments were repeated in triplicate under the same conditions
Data source location	Lomonosov Moscow State University, Moscow, Russia
Data accessibility	Raw data of the RNA-Seq are available in the Gene Expression Omnibus (GEO) – GSE180581 (https://www.ncbi.nlm.nih.gov/geo/query/acc.cgi?acc=GSE180581)

## Value of the Data

• These data are important for the investigation of transcriptional changes under Ku70, Ku80, or DNA-PKcs depletion in the cell.
• These data can be used by investigators of cellular functions of the DNA-PK complex and its individual subunits.
• These transcriptome data can be used to elucidate the independent cellular functions of Ku70, Ku80, and DNA-PKcs.

## 1 Data Description

Here we present transcriptomic analysis of HEK293T cells treated with control siRNA (293T + siCtr), HEK293T with monoallelic knockout of Ku70 [1,2] treated with siCtr (293TΔKu70 + siCtr) or siKu70 (293TΔKu70 + siKu70), HEK293T with monoallelic knockout of Ku80 [1] treated with siCtr (293TΔKu80 + siCtr) or siKu80 (293TΔKu80 + siKu80), and HEK293T with monoallelic knockout of DNA-PKcs subunit [1] treated with siCtr (293TΔDNA-PKcs + siCtr) or siDNA-PKcs (293TΔDNA-PKcs + siDNA-PKcs) in triplicate (Table 1). Transcriptomic data for each of 21 samples were obtained by RNA sequencing using an Illumina HiSeq 1500 platform. The raw reads are available at NCBI Biorepository: GEO accession ID GSE180581.

**Table 1:** General overview of samples described in this work. Short names are used to easily link data in Supplementary file 1 and main text.

Short name	Sample type	Cell type	Gene knockdown	Additional treatment	Repeat
G_A01	293T + siCtr	293T	None	50 nM siCtr	1
G_A02	293T + siCtr	293T	None	50 nM siCtr	2
G_A03	293T + siCtr	293T	None	50 nM siCtr	3
G_A04	293TΔKu70 + siCtr	293T	Ku70	50 nM siCtr	1
G_A05	293TΔKu70 + siCtr	293T	Ku70	50 nM siCtr	2
G_A06	293TΔKu70 + siCtr	293T	Ku70	50 nM siCtr	3
G_A07	293TΔKu70 + siKu70	293T	Ku70	25 nM siKu70_1 + 25 nM siKu70_2	1
G_A08	293TΔKu70 + siKu70	293T	Ku70	25 nM siKu70_1 + 25 nM siKu70_2	2
G_A09	293TΔKu70 + siKu70	293T	Ku70	25 nM siKu70_1 + 25 nM siKu70_2	3
G_A10	293TΔKu80 + siCtr	293T	Ku80	50 nM siCtr	1
G_A11	293TΔKu80 + siCtr	293T	Ku80	50 nM siCtr	2
G_A12	293TΔKu80 + siCtr	293T	Ku80	50 nM siCtr	3
G_A13	293TΔKu80 + siKu80	293T	Ku80	25 nM siKu80_1 + 25 nM siKu80_2	1
G_A14	293TΔKu80 + siKu80	293T	Ku80	25 nM siKu80_1 + 25 nM siKu80_2	2
G_A15	293TΔKu80 + siKu80	293T	Ku80	25 nM siKu80_1 + 25 nM siKu80_2	3
G_A16	293TΔDNA-PKcs + siCtr	293T	DNA-PKcs	50 nM siCtr	1
G_A17	293TΔDNA-PKcs + siCtr	293T	DNA-PKcs	50 nM siCtr	2
G_A18	293TΔDNA-PKcs + siCtr	293T	DNA-PKcs	50 nM siCtr	3
G_A19	293TΔDNA-PKcs + siDNA-PKcs	293T	DNA-PKcs	50 nM siDNA-PKcs	1
G_A20	293TΔDNA-PKcs + siDNA-PKcs	293T	DNA-PKcs	50 nM siDNA-PKcs	2
G_A21	293TΔDNA-PKcs + siDNA-PKcs	293T	DNA-PKcs	50 nM siDNA-PKcs	3

**Fig. 1:**
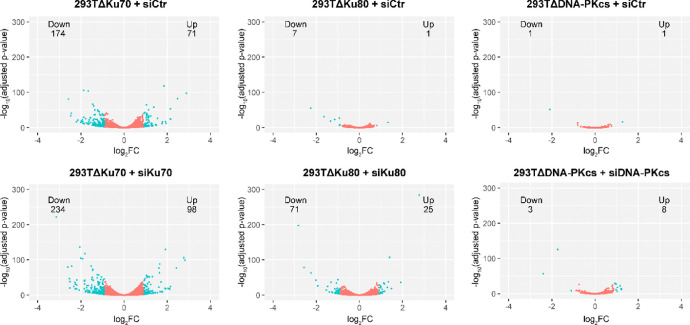
Volcano plots of differentially expressed genes in 293TΔKu70 + siCtr, 293TΔKu70 + siKu70, 293TΔKu80 + siCtr, 293TΔKu80 + siKu80, 293TΔDNA-PKcs + siCtr, 293TΔDNA-PKcs + siDNA-PKcs versus 293T + siCtr. The cyan dots represent genes for which log2FC < -0.9 or log2FC > 0.9 and FDR < 0.05. The numbers of up- and downregulated genes are also shown on the plots. The volcano plots were created using data described in Supplementary file 1.

Data presented in this article show genes differentially expressed in the cells depleted of Ku70 (293TΔKu70 + siCtr or 293TΔKu70 + siKu70), Ku80 (293TΔKu80 + siCtr or 293TΔKu80 + siKu80) or DNA-PKcs (293TΔDNA-PKcs + siCtr or 293TΔDNA-PKcs+ siDNA-PKcs) subunits of the DNA-PK complex and in the control cell line 293T + siCtr (Supplementary File 1). Volcano plots demonstrate the differentially expressed genes for each type of samples versus the control cell line (Fig. 1). We observed 174 down- and 71 upregulated genes in 293TΔKu70 + siCtr, 234 down- and 98 upregulated genes in 293TΔKu70 + siKu70, 7 down- and 1 upregulated genes in 293TΔKu80 + siCtr, 71 down- and 25 upregulated genes in 293TΔKu80 + siKu80, 1 down- and 1 upregulated genes in 293TΔDNA-PKcs + siCtr, 3 down- and 8 upregulated genes in 293TΔDNA-PKcs + siDNA-PKcs (Fig. 1).

## 2 Experimental Design, Materials and Methods

### 2.1 Cell cultures

HEK293T cells were cultured in DMEM medium supplemented with 10% FBS and 100 I.U./mL penicillin/100 µg/mL streptomycin solution (all purchased from Invitrogen). Preparation of HEK 293T cells with monoallelic knockout of Ku70, Ku80 and DNA-PKcs genes (Ku70+/-, Ku80+/- and DNA-PKcs+/-) was described in [1,2].

**Table 2:** List of siRNA used in the study.

siRNA name	siRNA strand	Sequence, 5’-3’	siRNA position in mRNA coding sequence
siCtr	sense	AGGUCGAACUACGGGUCAAdTsdT	NA
	antisense	UUGACCCGUAGUUCGACCUdTsdT	
siKu70_1	sense	GUGCAAAACGAAUUCUAGAdTsdT	352
	antisense	UCUAGAAUUCGUUUUGCACdTsdT	
siKu70_2	sense	GCUAAAACGGUUUGAUGAUdTsdT	1025
	antisense	AUCAUCAAACCGUUUUAGCdTsdT	
siKu80_1	sense	ACAAGGAUGAGAUUGCUUUdTsdT	179
	antisense	AAAGCAAUCUCAUCCUUGUdTsdT	
siKu80_2	sense	CAUGGGAAAUCAAGUUCUAdTsdT	423
	antisense	UAGAACUUGAUUUCCCAUGdTsdT	
siDNA-PKcs	sense	СUAUGAAACUACUGAAGGAdTsdT	9781
	antisense	UCCUUCAGUAGUUUCAUAGdTsdT	

### 2.2 Transient knock-down

To transfect cells with siRNAs, 50 pmol of pre-annealed siRNA duplex (for siKu70 25 pmol of siKu70_352 duplex and 25 pmol of siKu70_1025 duplex, for siKu80 25 pmol of siKu80_179 duplex and 25 pmol of siKu80_423 duplex) in OptiMem transfection medium were mixed with Lipofectamine RNAiMAX reagent (Invitrogen) according to the manufacturer's protocol and then added to 293T cells seeded in a 12-well plate in 1 mL of growth medium for 72 h incubation. The used siRNAs are listed in Table 2.

### 2.3 Total RNA Illumina sequencing

Total RNA fraction from all samples (3 repeats of HEK 293T wt independently transfected with siC, ΔKu70 with siKu70, ΔKu80 with siKu80 and ΔDNA-PKcs transfected with siDNA-PKcs) was extracted using TRIzol reagent (Life Technologies) following the manufacturer's protocol 72h after siRNA transfection. 1 µg of total RNA was fragmented by incubation at 90°C for 6 min in 40 µL of 100 mM Tris-HCl pH 8.0 and 8 mM MgCl_2_. After isopropanol precipitation, the RNA fraction was dissolved in 12 µL and subjected to ribosomal RNA depletion by NEBNext rRNA depletion kit (E6310L, New England Biolabs) according the manufacturer's protocol. mRNA sequencing libraries were prepared by NEBNext Ultra II Directional RNA library Prep kit for Illumina (E7760S, New England Biolabs) according to the manufacturer's protocol. NEBNext Multiplex Oligos for Illumina (Dual Index Primers Set1) (E7600S, New England Biolabs) were used for adaptor ligation. Quality control of the libraries was carried out on Agilent 2100 Bioanalyzer (Agilent Technologies). The libraries were sequenced on HiSeq 1500 platform (Illumina) at JSC Genoanalytica (Moscow, Russia).

### 2.4 Bioinformatic analysis

Quality of raw sequencing reads was checked by FastQC. Because of good quality of the reads they were mapped on genome without trimming. Raw sequencing data were mapped on hg38 human genome by STAR with default parameters. STAR was used for read counting for human genes obtained from the Ensemble database. Read coverage was normalized and compared among the samples using DESeq2 R library. Three independently transfected cell lines and the control cell line were compared. Negative binomial generalized linear models were used for differential expression analysis. We calculated the p-values adjusted on multiple testing (Benjamini and Hochberg method) and the logarithm of fold change of expression value (logFC) for all genes presented in the dataset (Supplementary file 1). The obtained data were visualized in R v. 3.6.0 as volcano-plots using ggplot2 library.

## Ethics Statement

Human subjects research: Not applicable.

Animal experiments: Not applicable.

Social media platforms: Not applicable.

## CRediT Author Statement

**Andrey Anisenko:** Investigation, Data analysis, Writing – original draft; **Olga Shadrina:** Data analysis, Writing – original draft; **Irina Garanina:** Data analysis; **Marina Gottikh:** Conceptualization, Supervision, Writing – review & editing.

## Funding

This work was supported by RSF grants 19-74-10021 (identification of differentially expressed genes in DNA-PKcs depleted cells) and 17-14-01107 (identification of differentially expressed genes in Ku70 and Ku80 depleted cells)

## Declaration of Competing Interest

The authors declare that they have no known competing financial interests or personal relationships which have or could be perceived to have influenced the work reported in this article.

## Appendix Supplementary materials

**Supplementary file 1.** Table of differentially expressed genes in 293TΔKu70 + siCtr, 293TΔKu70 + siKu70, 293TΔKu80 + siCtr, 293TΔKu80 + siKu80, 293TΔDNA-PKcs + siCtr, 293TΔDNA-PKcs + siDNA-PKcs versus 293T + siCtr. Image, application 1
